# Restructuring the front lines: how outpatient and community-based care is reorganizing rural healthcare

**DOI:** 10.1093/haschl/qxag297

**Published:** 2026-10-23

**Authors:** Anders Van Sandt, Anja Gruber, Craig Carpenter, Cris Meier, Scott Loveridge

**Affiliations:** Department of Agricultural and Resource Economics, Colorado State University, Fort Collins, CO 80523, United States; Department of Agricultural and Applied Economics, University of Wyoming, Laramie, WY 82071, United States; Department of Agricultural, Food, and Resource Economics, Michigan State University, East Lansing, MI 48824, United States; Department of Social Work, Utah State University, Logan, UT 84322, United States; Department of Agricultural, Food, and Resource Economics, Michigan State University, East Lansing, MI 48824, United States

**Keywords:** healthcare organization, rural, outpatient

## Abstract

**Introduction:**

Over the past 4 decades, US healthcare shifted from hospital-based to outpatient and community-based care, fundamentally changing frontline healthcare delivery.

**Methods:**

Using establishment-level administrative data from the Longitudinal Business Database (1980-2023) and the American Community Survey, we examine long-run trends across 9 healthcare industries in metro, metro-adjacent, and rural counties. We compare establishments per capita, employment location quotients, and consolidation, and estimate 2-way fixed effects models relating provider presence to payer mix, shortage-area designations, and socioeconomic conditions.

**Results:**

Hospitals and physician offices declined, while outpatient services expanded at different rates across industries and geography. Rural healthcare is not simply declining but is being reconfigured into a different mix of services, with greater reliance on outsourced care, especially for capital-intensive diagnostics. Despite outpatient growth, relative healthcare employment concentration remains lower across several frontline services in nonmetro counties. Provider presence is closely tied to payer mix, though associations vary across metro and nonmetro counties and service types.

**Conclusion:**

These findings suggest the transition toward outpatient care has produced distinct patterns of frontline healthcare availability across rural and urban counties.

## Introduction

The American healthcare system faces a persistent paradox. It is the most expensive in the world, costing $5.3 trillion in 2024 (18% of GDP), yet generates comparatively poor health outcomes, with adult and infant mortality exceeding peer nations.^1^ Within the United States, a second, rural mortality gap is continuing to widen even after controlling for race, ethnicity, education and income.^2^ These persistent disparities are consistent with what Probst et al. term “structural urbanism”: healthcare markets and policies tend to favor urban areas, leaving rural systems to contend with low patient volumes, challenging payer mixes, long travel distances, workforce recruitment and retention difficulties, and hospital closures.^3^ The study period (1980-2023) spans rapid technological change and major reforms to healthcare payment and delivery, including Medicare IPPS (1983),^4^ the Critical Access Hospital program (1997),^5^ home health payment reforms in 2000 (ref. 6) and 2014,^7^ and implementation of the Affordable Care Act (ACA)'s major coverage provisions and Medicaid expansion in 2014.^8^

While hospital closures and consolidation have received considerable attention, much less is known about how these broader structural changes have reshaped *frontline healthcare services*—the mix of healthcare providers through which communities access routine and ongoing healthcare.^9-11^ We use 40 years of restricted administrative data to examine how outpatient and community-based care has transformed the healthcare establishment landscape across rural and urban America. We compare healthcare establishments, employment patterns, and firm concentration before examining how local socioeconomic conditions relate to provider presence.

Across all county types, most outpatient and community-based healthcare services have expanded, while hospitals and physician offices have declined, with especially large declines in rural physician offices. These shifts in establishments are reflected in employment, where rural counties have lower relative employment concentrations in several frontline services despite growth in outpatient care. We also document increasing establishment size and firm concentration but find no evidence that consolidation differs across rural and urban counties. Finally, provider presence is closely associated with local payer mix, although these associations vary across metro and nonmetro counties and service types. Together, these findings suggest that rural healthcare is not simply declining but being reconfigured through a changing mix of services.

To our knowledge, this is the first national study to track the extensive margin of frontline healthcare availability (the presence and geographic distribution of healthcare establishments) alongside employment and ownership changes over four decades. These administrative data enable rural–urban comparisons unavailable from public business data or healthcare sources limited to particular providers or payers.

## Data and methods

We use the restricted-access Longitudinal Business Database (LBD), an administrative establishment-level dataset constructed from Census Bureau business records, economic censuses, and federal tax records. The LBD covers US private-sector employer establishments and provides establishment counts, employment, industry classifications, locations, and firm identifiers from 1980 to 2023. Unlike other data sources, including Medicare Provider of Services data, the LBD consistently measures establishments, employment, and common ownership across finely disaggregated industries without rural suppression, noise infusion, imputation, or restriction to Medicare-participating providers. Firm identifiers link individual physical business establishments, or locations, under common ownership, allowing us to track changes in the number of establishments operated by a firm. Firm size is measured by the total number of healthcare establishments operated across the sector, regardless of service type. To our knowledge, the LBD is the only source that applies a common administrative establishment definition across all included healthcare industries without rural suppression. Prior research comparing public and restricted establishment data documents substantial discrepancies in rural counties, making public provider counts an unsuitable benchmark for validating LBD establishment counts.^12^

Our empirical analyses examine hospitals alongside 8 outpatient and community-based healthcare industries: physician offices, mental health practices, freestanding ambulatory centers, home health services, laboratories and diagnostic centers, dialysis centers, outpatient mental health and substance abuse centers, and other outpatient care centers.^13^ The analysis spans selected frontline industries but does not encompass every institutional, residential, or specialized healthcare setting. Appendix Table S1 reports each industry's definition and first consistently identifiable year, accounting for classification changes including the transition from Standard Industrial Classification (SIC) codes to the North American Industry Classification System (NAICS). We examine 4 metrics: establishments per capita, location quotients (LQs), sector consolidation, and county-level provider presence.

Using the 2023 Rural–Urban Continuum Codes (RUCCs), we classify counties as metro (codes 1-3), metro-adjacent nonmetropolitan (4, 6, and 8), and nonadjacent rural (5, 7, and 9). Codes 1-3 distinguish metro counties by metropolitan area size, while the nonmetro codes distinguish counties by urban population and adjacency to a metro county. We apply this fixed classification throughout the study to avoid conflating healthcare trends with counties moving between categories across RUCC vintages. We distinguish adjacent from nonadjacent nonmetro counties because proximity to a metro area shapes access to regional referral networks and healthcare labor markets. Restricted annual American Community Survey (ACS) covariates include uninsured and Medicaid enrollment rates, the share aged 65 or older (a proxy for Medicare), educational attainment, and median family income. Health Professional Shortage Area (HPSA) designations come from the Area Health Resources Files (AHRF).

We first compare trends in establishment counts per capita, employment LQs, and industry consolidation across county ruralities. Establishments represent nodes of service presence, the extensive margin of availability, rather than capacity, service mix, or workforce composition. We then estimate separate industry-specific two-way fixed effects (TWFE) models for metro and nonmetro counties from 2005 to 2022, using county-year establishment counts as the outcome. Consistent with demand-threshold approaches, log population accounts for market size. Covariates come from the restricted annual ACS, annual Census population estimates, and AHRF primary care and mental health HPSA indicators. The unweighted models include county and year fixed effects, with standard errors clustered at the county level. (Additional methodological details are available in the Appendix.) Our objective is to characterize associations with local conditions rather than identify causal effects.

## Results

### A shift toward outpatient care

Since 1980, frontline healthcare has become increasingly centered on outpatient and community-based settings as care has shifted away from hospitals and independent physician offices. Figure 1 shows establishments per 10 000 residents for hospitals, physician offices, and outpatient services, which include laboratories and diagnostic centers beginning in 1980, adds dialysis centers and home health services in 1992, and includes all of our frontline healthcare industries from 1997 onward. The composite incorporates industries as they become consistently identifiable, so changes around their entry years may reflect composition as well as growth; Appendix Figure S1 reports the industry-specific trends.

**Figure 1.**
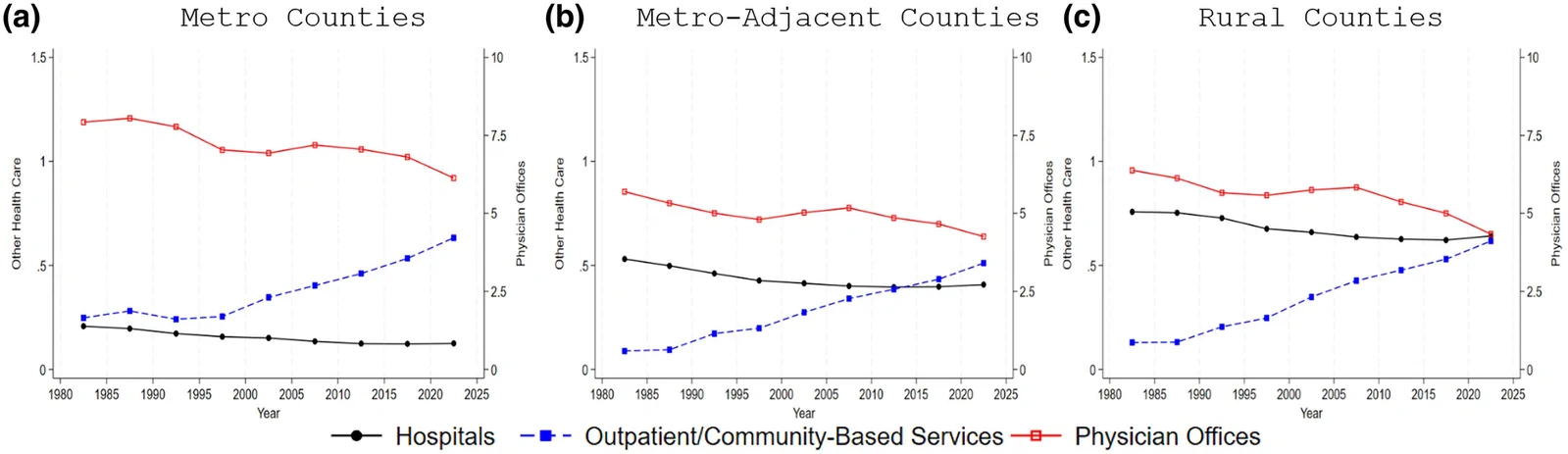
Establishments per 10 000 population over time. Source: Authors’ analysis of data from the Longitudinal Business Database, 2026. The figure reports unweighted averages of county-level establishments per 10 000 residents in (a) metro (2023 USDA RUCC 1-3), (b) metro-adjacent (RUCC 4, 6, and 8), and (c) rural (RUCC 5, 7, and 9) counties. For each county, the rate is calculated by dividing the 5-year average establishment count by the 5-year average population and multiplying by 10 000. County-years with no establishments are retained as zeros, and 5-year averaging was required for disclosure. Industries are shown beginning when consistently identifiable in the 1980-2023 LBD. The outpatient/community-based measure averages establishment counts across the included industries identifiable in each period. The Census Bureau has reviewed this data product to ensure appropriate access, use, and disclosure avoidance protection of the confidential source data. Research was performed at a Federal Statistical Research Data Center under FSRDC Project Number 2716, clearance request #13037.

Hospital concentration declined across urban, metro-adjacent, and rural counties over the study period. Rural counties maintained higher hospital concentrations, but still experienced a 15% decline, compared to declines of 23% and 40% in metro-adjacent and urban counties, respectively.

While hospital decline has received considerable attention in the literature, physician offices experienced the most pronounced contraction, particularly in rural areas. Rural physician office concentration declined 32% to 4.3 establishments per 10 000 residents, bringing it in line with metro-adjacent counties (4.26 after a 25% decline), both of which now lag urban counties (6.1 after a 23% decline). Over the past 4 decades, rural physician office concentration fell more than twice as much as rural hospital concentration.

In contrast, every outpatient and community-based service that we considered expanded, with interruptions in home health growth. Freestanding ambulatory centers (including urgent care centers) saw the fastest growth, increasing by more than 500% in rural and metro-adjacent counties, although they remain more concentrated in urban areas (Appendix Figure S1). All Other Outpatient Centers, which include Federally Qualified Health Centers (FQHCs) and Rural Health Clinics, quadrupled in rural and metro-adjacent counties since 1997, reflecting their growing role as a safety net in underserved communities. This is consistent with prior work showing that FQHCs are more likely to locate near recently closed rural hospitals.^14^ Diagnostic labs also increased but remain disproportionately concentrated in urban counties, with roughly twice the establishments per capita as rural counties. Mental health offices grew faster in rural and metro-adjacent counties than in urban counties, yet their overall concentration remains roughly half that of urban counties. In contrast, outpatient mental health and substance abuse centers are more concentrated in rural counties, reflecting both local need and targeted funding programs. Home health services were the third-fastest-growing industry, behind dialysis centers and freestanding ambulatory centers, and maintain the highest establishment rate in rural counties. Growth was similar across geographies until 2012, when growth stagnated in metro-adjacent and rural counties (Appendix Figure S1). These findings suggest rural healthcare is being restructured rather than uniformly reduced.

### Rural America has a different healthcare service mix

Given the changing composition of healthcare delivery, Figure 2 examines the distribution of healthcare employment across ruralities. An industry's LQ divides its share of county employment by its share of national employment; a value of more than one indicates that the industry is overrepresented in the local employment base (see Appendix Section 2 for additional details on this calculation). LQs describe the relative role of each healthcare industry in the local economy rather than service availability or capacity. In rural counties, high LQs may partly reflect thin local labor markets in which healthcare providers are major employers. We consequently interpret LQs as complementary to, rather than substitutes for, the establishment per capita measures.

**Figure 2.**
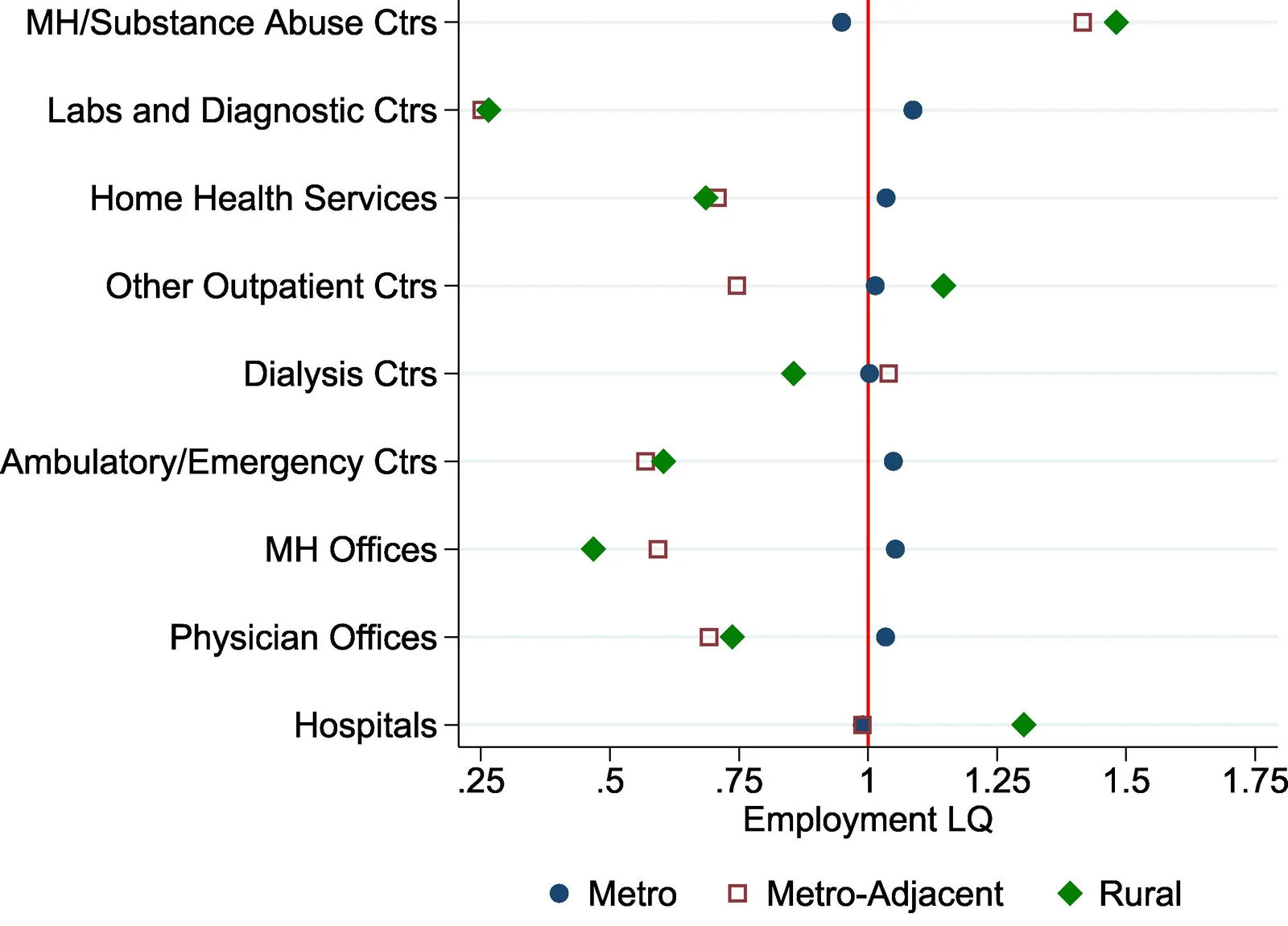
Healthcare sub-industry employment location quotients by type of county (2023). Source: Authors’ analysis of data from the Longitudinal Business Database, 2026.

As expected, urban counties have LQs near one across most industries, reflecting their share of national employment. In contrast, rural counties exhibit substantial variation, with higher relative employment concentrations in hospitals and outpatient mental health and substance abuse centers. Other outpatient care centers are also modestly overrepresented in rural employment, whereas they remain underrepresented in metro-adjacent counties. Rural counties have lower relative employment concentrations in physician offices, mental health offices, freestanding ambulatory centers, home health services, and diagnostic labs. The relative employment concentration of rural and metro-adjacent labs and diagnostic centers is approximately one-quarter that of urban counties, consistent with greater reliance on external diagnostic systems. Likewise, despite sustained attention and programs designed to expand rural mental healthcare, the rural employment concentration of mental health offices remains less than half that of urban counties.^15^

These employment patterns complement the establishment trends, showing that despite growth in outpatient establishments, employment remains unevenly distributed across healthcare services. As a result, these measures show that the mix of frontline establishments and healthcare employment differs across ruralities.

### Consolidation is changing ownership, but not rurality

The restructuring of frontline healthcare has occurred alongside substantial changes in ownership structure and firm organization. Metro and nonmetro counties exhibited similar consolidation trends (Figure 3), so we only disclose the total counts. Across the healthcare sector, average employees per establishment have increased steadily since 2008, indicating growing consolidation of labor within fewer, larger entities. The share of establishments owned by single-establishment firms has declined by more than 28% since 1980, while the share of healthcare establishments belonging to firms operating at least 5 establishments across the sector has grown nearly 9-fold.

**Figure 3.**
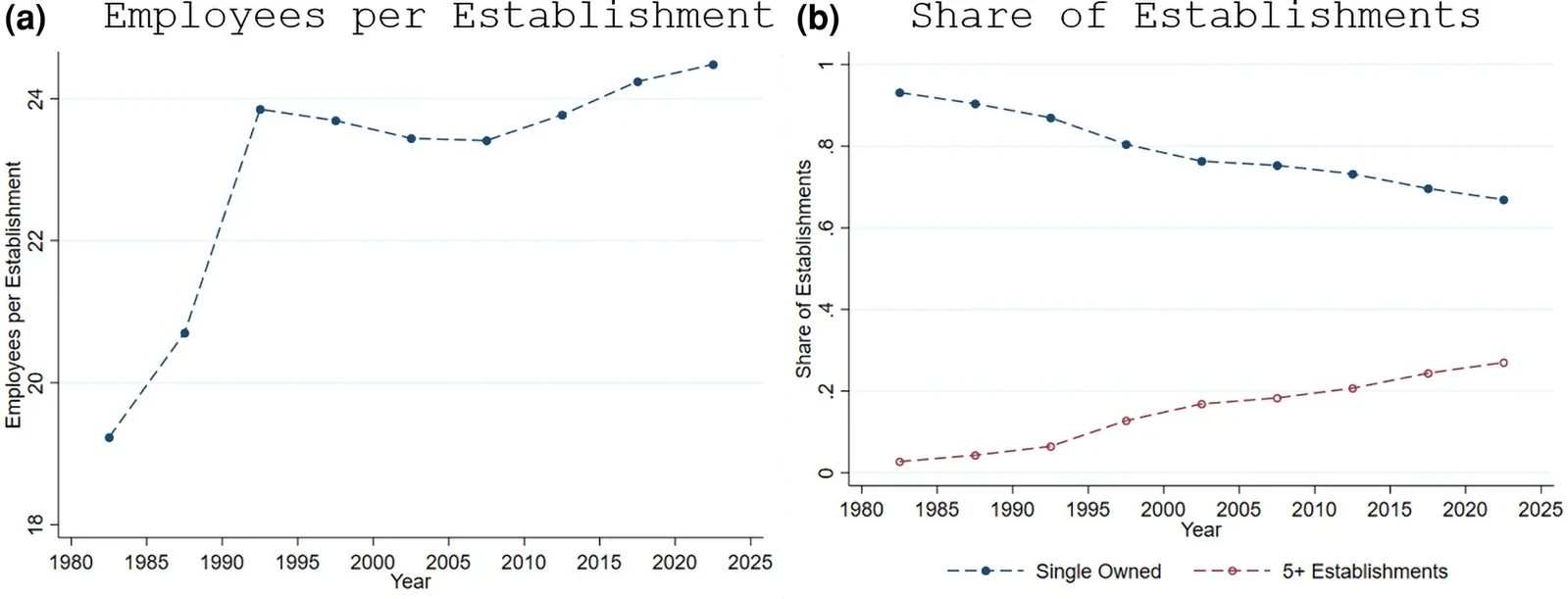
Healthcare industry consolidation over time in terms of (a) employees per establishment and (b) share of establishments that are single owned versus have 5+ establishments. Source: Authors’ analysis of data from the Longitudinal Business Database, 2026.

These trends are consistent with broader evidence of healthcare consolidation.^16^ Patterns were similar across rural, metro-adjacent, and urban counties, suggesting that consolidation is a system-wide phenomenon.

### Payer mix and structural constraints relate to provider presence

While consolidation describes how providers have become organized, understanding where providers locate requires examining the local economic and demographic conditions associated with provider presence. To better understand local factors associated with provider presence, we estimate county-by-year TWFE models relating healthcare establishments to county-level socioeconomic characteristics, payer mix, and HPSA designations over the 2005-2022 regression period. Figure 4 identifies several consistent patterns that differ between metro and nonmetro counties. Rural and metro-adjacent counties (RUCC 4-9) were aggregated in this section to streamline the analysis. Figure 4 emphasizes patterns in direction and statistical significance, as the count models are estimated separately by industry and county type and their magnitudes are not directly comparable. Coefficient estimates are reported in Appendix Table S2.

**Figure 4.**
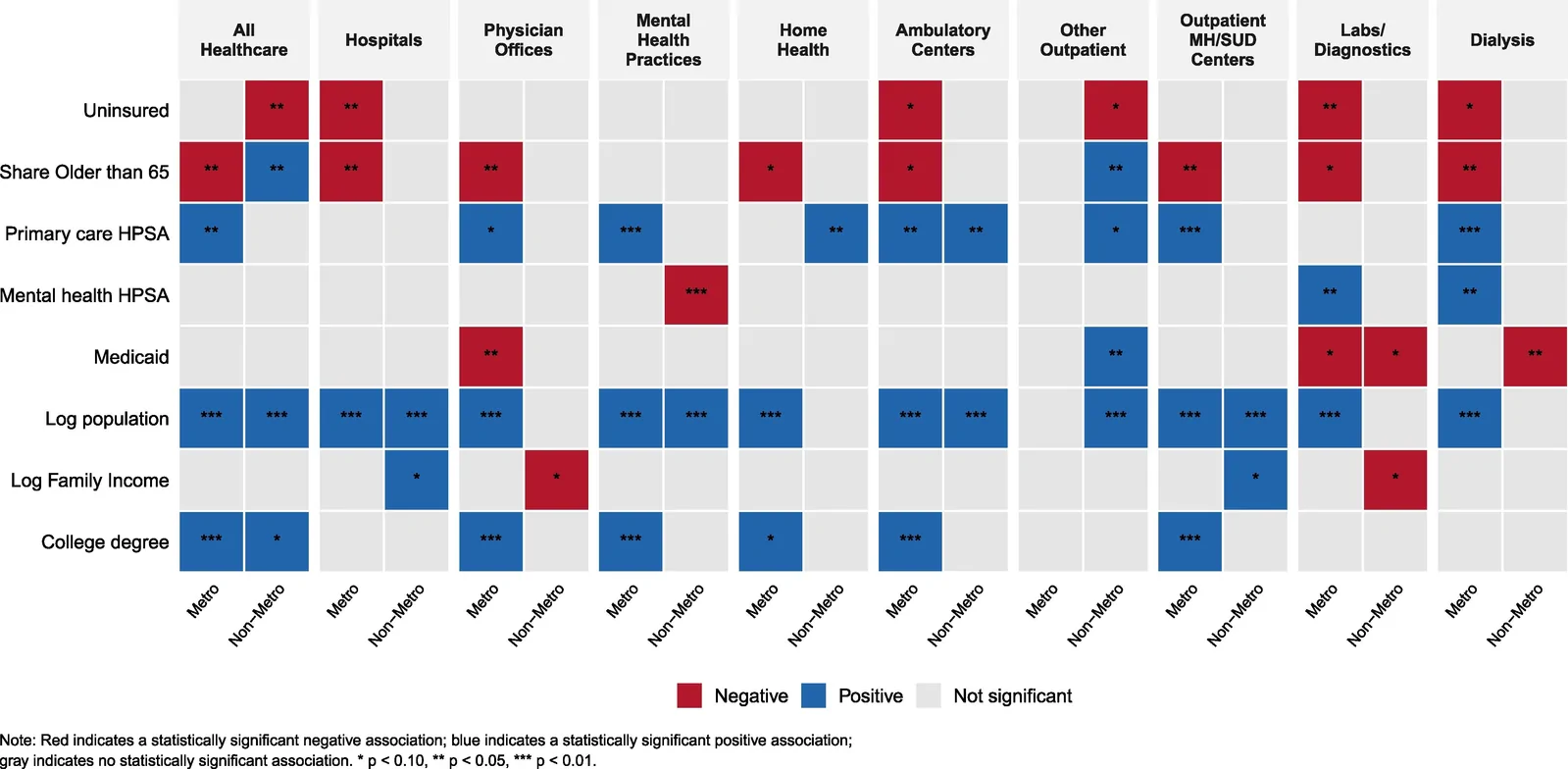
Heatmap between establishments and county-level covariates (TWFE). Source: Authors’ analysis of data from the Longitudinal Business Database and the American Community Survey, 2026. Red indicates a statistically significant negative association; blue indicates a statistically significant positive association; gray indicates no statistically significant association. *P < .10, **P < .05, ***P < .01.

Three patterns emerge. First, provider presence is strongly associated with payer mix. An increase in uninsured residents is associated with fewer healthcare establishments, particularly in metro counties, where increases in the uninsurance rate are associated with fewer hospitals, diagnostic labs, and marginally fewer freestanding ambulatory centers and dialysis services. Changes in the Medicare-eligible population (proxied by the share over age 65) show divergent patterns, with metro counties associated with fewer establishments across multiple services and nonmetro counties associated with more total healthcare establishments and more outpatient services. Increases in Medicaid shares are associated with outpatient center growth in nonmetro counties (likely FQHCs) but fewer physician offices in metro counties, fewer dialysis services in nonmetro counties, and marginally fewer labs and diagnostic centers in both. These patterns suggest that provider presence is closely tied to payer mix, though effects vary by geography and service type.

Second, HPSA designations exhibit varying associations across industries. Because designation partly reflects existing provider supply and eligibility for targeted programs, these estimates should be interpreted as descriptive associations rather than independent effects of workforce shortages. In metro counties, gaining Primary Care-Health Professional Shortage Area designation (PC-HPSA) is associated with increased healthcare establishments, physician offices, mental health services, freestanding ambulatory centers, dialysis centers, and substance abuse centers, as well as increased outpatient centers, home health services, and freestanding ambulatory centers in nonmetro counties. In contrast, gaining Mental Health-Health Professional Shortage Area (MH-HPSA) designation is associated with fewer mental health practices in nonmetro counties.

Third, local socioeconomic characteristics are associated with provider presence, although these relationships differ between metro and nonmetro counties. A higher college-educated population share is associated with more healthcare establishments overall, physician offices, mental health practices, outpatient mental health and substance abuse centers, and freestanding ambulatory centers in metro counties. It is also marginally associated with more healthcare establishments overall in nonmetro counties and more home health establishments in metro counties. Growth in family income is marginally associated with more hospitals and outpatient mental health centers in nonmetro counties, but fewer physician offices and diagnostic labs over time. Finally, sensitivity analyses using employment produced qualitatively similar industry trends over time and regression patterns. Given the number of industry-specific estimates, marginal associations (*P* < .10) should be interpreted cautiously.

## Discussion

Our findings suggest that the restructuring of frontline healthcare services extends beyond the movement of care from hospitals to outpatient settings. Over the past 4 decades, the mix of frontline healthcare establishments has changed as outpatient care expanded, provider ownership became more concentrated, and the availability of individual services diverged across places. These patterns point to a broader reorganization of the healthcare system.

Historically, healthcare services were delivered primarily through independent, locally embedded physician practices and community hospitals, with organizational and investment decisions largely made at the local level.^17,18^ Over time, this model has shifted toward multi-establishment systems: since 1980, the share of establishments owned by single-establishment firms has decreased 28%, while the share of healthcare establishments belonging to firms operating 5 or more healthcare establishments has grown nearly 9-fold. As providers become embedded within larger health systems, decisions regarding service provision and investment increasingly shift from the community to the health system, where capital is allocated across markets rather than within individual communities.^19^

Within this reorganized healthcare landscape, payer mix appears to function as a signal of financial viability and is associated with provider presence, consistent with a broader literature linking reimbursement mix to provider financial performance.^20^ Counties with higher uninsured rates frequently have fewer healthcare services, while higher shares of Medicaid beneficiaries are associated with mixed, but often fewer, establishments. These associations differ in sign and statistical significance across metro and nonmetro counties and service types, although our separately estimated models do not test whether they are weaker in nonmetro counties. Provider presence in nonmetro counties may also be shaped by infrastructure, workforce, and patient volume constraints that our models do not directly measure.^21^ These findings suggest healthcare investment and service provision decisions increasingly occur at the health-system level rather than within individual communities.

The transition toward outpatient and community-based care has unfolded differently across frontline healthcare services. Diagnostic labs remain disproportionately concentrated in urban counties despite overall growth, likely reflecting their increasingly capital-intensive nature and incentives to outsource diagnostics to centralized regional hubs that benefit from economies of scale. This interpretation is consistent with evidence that vertical integration shifts diagnostic imaging and laboratory services toward larger health systems and different sites of care.^22^ Outpatient centers, such as FQHCs, experienced some of the fastest growth, particularly in rural counties, reinforcing their growing role as safety-net providers. Home health expanded across geographies before stagnating in rural and metro-adjacent counties after 2012, potentially reflecting reimbursement changes, tight margins, and the higher costs of delivering care across dispersed populations.^7,21^ Freestanding ambulatory centers (eg, urgent care centers) experienced the most rapid growth but remain more concentrated in urban counties, suggesting that patient volume and local market conditions may continue to shape their distribution.

These findings suggest that communities are not simply losing providers but developing increasingly distinct mixes of frontline healthcare services. Although hospitals and physician offices declined, outpatient growth has varied across industries and places, leaving communities increasingly reliant on different combinations of providers to meet healthcare needs. The goal is not local provision of every frontline service, but access to coordinated care through providers operating across broader regional healthcare systems. Understanding how this service mix evolves, and the financial and structural conditions associated with provider presence, is critical to reducing geographic disparities in access to care.

## Policy implications

Much of rural healthcare policy has sought to maintain access to care by preserving rural hospitals and other traditional providers.^11^ While maintaining access remains the goal, the healthcare system reorganized toward outpatient and community-based care, resulting in distinct healthcare mixes across communities. Rather than assuming every community should retain its historical provider mix or host every service, policymakers should distinguish services requiring local presence from those sustainably delivered through regional referral networks. This perspective shifts attention from preserving individual facilities toward supporting coordinated and financially sustainable systems of care.

First, policies should consider how reimbursement systems shape geographic access to care. Provider presence is associated with payer mix, although our descriptive models do not identify the effects of specific payment policies. Future research should assess whether payment stability and adjustments for the higher costs and lower patient volumes of rural care support local service presence.

Second, workforce availability remains central to rural healthcare, but HPSA designations capture only shortages among designated primary care and mental health clinicians. They do not provide a comprehensive measure of the nurses, technicians, aides, administrative staff, and other workers needed to operate healthcare establishments. Our results therefore should not be interpreted as showing that workforce constraints are less important than financial or infrastructure constraints. Policies addressing rural workforce shortages should consider the full healthcare team and pair clinician-focused incentives with broader investments in the systems that support rural practice.

Third, policymakers should recognize that the restructuring of frontline healthcare extends well beyond hospitals. Much of rural healthcare policy has focused on preserving hospitals, expanding insurance coverage, or addressing workforce shortages. Outpatient and community-based providers are becoming an increasingly important component of the healthcare system and should receive similar policy attention. As care shifts outside hospitals and consolidation continues, policymakers should consider how reimbursement systems, provider consolidation, and capital investment shape the geographic distribution of frontline healthcare services. Focusing solely on hospitals risks overlooking broader changes in the organization of healthcare that increasingly determine where patients receive routine care.

## Limitations

This study is descriptive and more rigorous causal analyses using administrative data are needed to better understand the mechanisms and refine policy recommendations. While we examine establishment trends and employment concentrations, these measures capture the presence and organization of service nodes rather than facility capacity, service mix, workforce composition, quality, or realized access; a single establishment may therefore produce a high per-capita rate in a sparsely populated county despite limited capacity or reach. Because our analysis excludes skilled nursing facilities, we cannot assess substitution among hospitals, skilled nursing facilities, and home health services, an important area for future research. Finally, the population share age 65 and older is only a proxy for Medicare coverage, while HPSA designations are partly based on existing provider-to-population ratios, may trigger targeted policy supports, and do not capture the broader availability of nurses, technicians, aides, administrative staff, and other workers needed to operate healthcare establishments. In addition, because metro and nonmetro models are estimated separately using establishment counts on different scales, we do not formally test whether associations differ in strength across county types. Applying the 2023 RUCCs to all years also means that trends for each county type describe a fixed set of counties rather than counties’ contemporaneous classifications.

## Conclusions

The transition toward outpatient and community-based care has fundamentally reshaped the composition, geography, ownership, and incentives of the US healthcare system. Outpatient and community-based services expanded unevenly, changing the mix of frontline establishments across communities as ownership became more concentrated. Our findings suggest that provider presence is closely associated with payer mix and structural constraints, raising concerns that communities with fewer financial resources may face growing challenges maintaining frontline healthcare services. Addressing these disparities will require policies that move beyond workforce expansion and insurance coverage alone to directly address the broader restructuring of healthcare and its implications for access. These changes may also transform how patients experience healthcare: rather than relying on a single physician office or local hospital, patients may increasingly navigate multiple specialized providers. Understanding how this changing service mix affects care coordination, continuity, patient experience, and health outcomes represents an important direction for future research.

## Supplementary Material

qxag297_Supplementary_Data
