# Supplementary material for: Restructuring the front lines: how outpatient and community-based care is reorganizing rural healthcare
Source: Health Aff Sch. 2026 Oct 23;4(11):qxag297. doi: 10.1093/haschl/qxag297 (PMC13732678; doi:10.1093/haschl/qxag297)
Supplement: Supplementary file 1 [file qxag297_supplementary_data.zip › Supplemental_Material.docx]

**Online Appendix**

**Section 1: Detailed Data Description**

The main data in our analysis come from the Longitudinal Business Database (LBD), which we accessed through a Federal Statistical Research Data Center (FSRDC). The LBD cross-references economic census data with Internal Revenue Service business tax records to retime business births and deaths and allows us to comprehensively and accurately measure the number of points of availability of healthcare (“establishments”) independent of their insurance acceptance or enrollment, as well as the number of employees per establishment, and understand common ownership structures across establishments. The LBD provides a separate identifier for each establishment as well as for each firm, meaning that we can identify health care establishments with common ownership.

We combine our data with restricted-access American Community Survey (ACS) data from 2005-2022, which allows us to retain data for small rural counties that are not identified in the public-use version. We collapse the data to the county-by-year level and supplement the data with annual census county population estimates and indicators for mental health and primary care health professional shortage area (HPSA) designation from the Area Health Resources Files (AHRF) for the same time period.

**Section 2: Detailed Methodology**

a. **Location Quotient**

We calculate the location quotient in sub-industry i and county type c as

$LQ_{ic}= {\frac{E_{ic}}{E_{c}}}/{\frac{E_{i}}{E}}$,

where the numerator is sub-industry i’s share of employment in county type c and the denominator is its share of national employment. A location quotient equal to one indicates that employment in a specific health care sub-industry is proportional to the national share of employment in that sub-industry. Location quotients below 1 indicate that the share of employment in the sub-industry is underrepresented in a given county type; location quotients above 1 show that it is overrepresented. We rely on employment counts at the county-type by sub-industry level calculated from 2023 individual establishment LBD data to construct our location quotients.

b. **Healthcare Industry Consolidation over Time**

We calculate two separate measures of industry consolidation over time. First, we use 1980-2022 LBD data on the number of employees at individual establishments to calculate the average number of employees per establishment. We calculate 5-year averages to satisfy disclosure requirements. We calculate a second measure of consolidation that evaluates the ownership structure over time. We divide the number of establishments that are owned by a firm with only one healthcare establishment by the total number of health care establishments to determine the share of establishments that are “single owned”, and we divide the number of establishments that have a firm that owns at least 5 distinct health care establishments by the total number of health care establishments to calculate the share of establishments with “5+ Establishments”.

c. **TWFE Analysis**

Our regression analysis relies on county-by-year number of health care establishments from LBD data, as well as county-by-year covariates from the ACS, annual census county population estimates, and indicators for mental health and primary care health professional shortage area (HPSA) designation from the Area Health Resources Files (AHRF). Our analysis is restricted to the 2005-2022 time period, and we conduct separate analyses for the sample of metro counties (RUCC 1-3) and non-metro counties (RUCC 4-9) for each of the sub-industries of interest.

For each sub-industry and county type, we estimate the association between the number of health care establishments $E_{yc}$ and county-by-year covariates as

$E_{yc}= \beta X_{yc}+\alpha_{c}+\gamma_{y}+\varepsilon_{yc}$,

where the covariates $X_{yc}$include the share of a county’s population without health insurance, the share insured through Medicaid, the share of the population older than 65, the log median family income, the share of the population with a college degree, indicators for mental health (MH) and primary care (PC) HPSA designation, as well as log population. The unweighted models also include county fixed effects, α, and year fixed effects, γ. Standard errors are clustered at the county level.

**Section 3: Appendix Figures**

**Figure A1: Sub-Industry Health Care Establishments per Population**

**a) Dialysis Centers**

**d) Ambulatory & Emergency Centers**

**b) Home Health Services**

**e) Labs and Imaging Centers**

**g) Mental Health Offices**

**c) Other Outpatient Centers**

**f) MH & Substance Abuse Centers**

Source: Authors’ analysis of data from the Longitudinal Business Database, 2026.

*Notes*: Figure A1 reports unweighted averages of county-level establishments per 10,000 residents, calculated as in Figure 1, in metro (2023 USDA RUCC 1-3), metro-adjacent (2023 USDA RUCC 4, 6, 8), and rural (2023 USDA RUCC 5, 7, 9) counties in healthcare sub-industries when identifiable using data from the 1980-2023 LBD. The Census Bureau has reviewed this data product to ensure appropriate access, use, and disclosure avoidance protection of the confidential source data. Research was performed at a Federal Statistical Research Data Center under FSRDC Project Number 2716, clearance request #13037.

**Section 4: Appendix Tables**

**Table A1: Healthcare Industries and Definitions**

| **Industry** | **SIC (1980–1997)** | **NAICS (1997–2023)** | **Description** |
| --- | --- | --- | --- |
| Hospitals | 8062 | 6221 | General medical and surgical hospitals |
| Physician Offices | 801 | 6211 | Offices of Physicians (except Mental Health Specialists) |
| Mental Health Practices |  | 621112, 621330 | Offices of Physicians - Mental Health Specialists, Offices of Mental Health Practitioners (except physicians) |
| Freestanding Ambulatory Centers |  | 621493 | Freestanding Ambulatory Surgical and Emergency Centers |
| Home Health Services | 8082 | 621610 | Home Health Care Services |
| Laboratories & Diagnostic Centers | 8071 | 62151 | Medical and Diagnostic Laboratories |
| Dialysis Centers | 8092 | 621492 | Kidney Dialysis Centers |
| Outpatient Mental Health & Substance Abuse Centers |  | 621420 | Outpatient Mental Health and Substance Abuse Centers |
| Other Outpatient Care Centers |  | 621498 | All Other Outpatient Care Centers |

Source: North American Industry Classification System, U.S. Census Bureau, 2026. First consistently identifiable year: hospitals, physician offices, and laboratories and diagnostic centers, 1980; home health services and dialysis centers, 1992; all other industries, 1997.

**Table A2: Associations between Establishments and County-Level Covariates (TWFE)**

|  | **All Health Care** | | **Hospitals** | | **Physician Offices** | | **Mental Health Practices** | | **Freestanding Ambulatory Ctrs** | |
| --- | --- | --- | --- | --- | --- | --- | --- | --- | --- | --- |
|  | **Metro** | **Non-Metro** | **Metro** | **Non-Metro** | **Metro** | **Non-Metro** | **Metro** | **Non-Metro** | **Metro** | **Non-Metro** |
| Share No | -5019 | -64.08** | -1012** | -19.17 | -534.6 | 8.954 | -228.6 | -0.383 | -68.63* | 0.006 |
| Health Insurance | (3298) | (26.85) | (488) | (11.94) | (455.8) | (7.539) | (162) | (1.278) | (35.74) | (1.455) |
|  |  |  |  |  |  |  |  |  |  |  |
| Share Medicaid | -135.6 | 30.01 | 59.65 | 17.09 | -344.1** | -9.855 | 45.81 | 1.698 | -15.91 | -0.503 |
|  | (2282) | (24.82) | (286.2) | (10.63) | (149.1) | (9.376) | (79.06) | (1.206) | (28.30) | (1.382) |
|  |  |  |  |  |  |  |  |  |  |  |
| Share Older | -4523** | 53.80** | -919.8** | 14.92 | -672.7** | 4.416 | -95.35 | 0.098 | -58.26* | 0.200 |
| than 65 | (2018) | (21.15) | (428.0) | (11.99) | (303.9) | (4.618) | (79.70) | (1.274) | (31.95) | (1.377) |
|  |  |  |  |  |  |  |  |  |  |  |
| Log Family Income | -95.42 | 5.043 | 160.0 | 6.112* | -88.36 | -4.425* | 44.67 | 0.466 | -8.028 | -0.18 |
|  | (1039) | (6.845) | (125.7) | (3.618) | (96.86) | (2.403) | (32.56) | (0.325) | (7.615) | (0.253) |
|  |  |  |  |  |  |  |  |  |  |  |
| Share with | 8513*** | 45.02* | 889.8 | 6.533 | 1197*** | 5.753 | 385.8*** | -0.713 | 119.4*** | -1.700 |
| College Degree | (2806) | (25.31) | (535.0) | (15.26) | (335.4) | (10.71) | (119.8) | (1.344) | (43.26) | (1.345) |
|  |  |  |  |  |  |  |  |  |  |  |
| PC HPSA | 1138** | 2.778 | 107.4 | -2.489 | 97.05* | -1.592 | 30.36*** | 0.523 | 16.98** | 0.706** |
| Designation | (426.9) | (6.430) | (68.54) | (4.347) | (51.97) | (1.567) | (10.38) | (0.445) | (7.377) | (0.278) |
|  |  |  |  |  |  |  |  |  |  |  |
| MH HPSA | 409.5 | 13.12 | 58.73 | 0.148 | 36.05 | 0.479 | 8.095 | -2.175*** | 9.950 | -0.136 |
| Designation | (351.5) | (9.801) | (50.21) | (5.549) | (34.14) | (2.997) | (13.07) | (0.794) | (5.967) | (0.589) |
|  |  |  |  |  |  |  |  |  |  |  |
| Log Population | 16840*** | 684.5*** | 2849*** | 175.9*** | 2664*** | 8.241 | 487.1*** | 19.14*** | 297.8*** | 11.79*** |
|  | (2070) | (141.0) | (348.8) | (62.80) | (376.4) | (33.97) | (63.97) | (4.81) | (68.75) | (4.148) |

**Table A2 (continued)**

|  | **Dialysis Ctrs** | | **Other Outpatient Ctrs** | | **Home Health Services** | | **Labs and Diagnostic Ctrs** | | **Outpatient MH and Substance Abuse Ctrs** | |
| --- | --- | --- | --- | --- | --- | --- | --- | --- | --- | --- |
|  | **Metro** | **Non-Metro** | **Metro** | **Non-Metro** | **Metro** | **Non-Metro** | **Metro** | **Non-Metro** | **Metro** | **Non-Metro** |
| Share No | -82.55* | 0.482 | 890.5 | -8.069* | -188.8 | -9.190 | -168.2** | -1.206 | -87.67 | 2.035 |
| Health Insurance | (49.04) | (0.683) | (1202) | (4.478) | (344.0) | (8.111) | (75.4) | (1.148) | (64.48) | (3.246) |
|  |  |  |  |  |  |  |  |  |  |  |
| Share Medicaid | 17.16 | -1.306** | -1284 | 9.955** | 721.4 | 10.12 | -93.26* | -1.439* | 50.26 | 2.495 |
|  | (23.79) | (0.619) | (1504) | (4.908) | (516.2) | (9.174) | (50.81) | (0.827) | (41.62) | (2.170) |
|  |  |  |  |  |  |  |  |  |  |  |
| Share Older | -81.82** | 1.634 | 318.0 | 8.731** | -540.7* | 6.611 | -99.39* | 0.334 | -130.3** | 0.355 |
| than 65 | (32.56) | (1.768) | (593.6) | (4.086) | (303.4) | (8.97) | (49.59) | (0.769) | (64.58) | (2.198) |
|  |  |  |  |  |  |  |  |  |  |  |
| Log Family Income | -0.739 | 0.249 | -846.0 | 2.280 | 149.0 | 0.447 | 7.576 | -0.420* | 17.13 | 0.982* |
|  | (6.069) | (0.187) | (931.5) | (1.372) | (151.4) | (0.980) | (19.59) | (0.231) | (15.61) | (0.561) |
|  |  |  |  |  |  |  |  |  |  |  |
| Share with | 37.87 | 1.085 | 855.4 | 10.77 | 1635* | -3.379 | 147.4 | 0.109 | 215.5*** | 1.045 |
| College Degree | (31.00) | (1.944) | (552.1) | (8.248) | (949.6) | (7.957) | (94.0) | (0.840) | (70.08) | (2.066) |
|  |  |  |  |  |  |  |  |  |  |  |
| PC HPSA | 15.95*** | -0.015 | 325.5 | 1.958* | 94.57 | 3.497** | 17.32 | 0.274 | 25.31*** | 0.867 |
| Designation | (4.841) | (0.316) | (278.3) | (1.138) | (62.27) | (1.431) | (11.29) | (0.192) | (9.004) | (0.758) |
|  |  |  |  |  |  |  |  |  |  |  |
| MH HPSA | 8.173** | 0.476 | -149.0 | 2.858 | 53.73 | -5.522 | 28.78** | -0.528 | 8.88 | 1.421 |
| Designation | (3.519) | (0.613) | (173.2) | (2.970) | (40.57) | (4.052) | (12.35) | (0.439) | (7.71) | (1.253) |
|  |  |  |  |  |  |  |  |  |  |  |
| Log Population | 85.87*** | 12.09 | 1430 | 68.42*** | 1305*** | 11.53 | 413.9*** | 1.095 | 308.0*** | 33.83*** |
|  | (24.69) | (11.03) | (1080) | (15.53) | (235.7) | (36.23) | (86.81) | (2.480) | (82.73) | (11.09) |
|  |  |  |  |  |  |  |  |  |  |  |
| Observations | 17500 | 29000 | 17500 | 29000 | 17500 | 29000 | 17500 | 29000 | 17500 | 29000 |

Source: Authors’ analysis of data from the 2005-2022 Longitudinal Business Database and the American Community Survey, 2026. Observation counts are rounded in accordance with disclosure rules. Standard errors clustered at the county level are in parentheses. * (p<0.10), ** (p<0.05), *** (p<0.01).
